# Ritonavir reverses resistance to docetaxel and cabazitaxel in prostate cancer cells with acquired resistance to docetaxel

**DOI:** 10.20517/cdr.2023.136

**Published:** 2024-01-31

**Authors:** Eric van der Putten, Katja Wosikowski, Jos H. Beijnen, Gábor Imre, Colin R. Freund

**Affiliations:** ^1^Modra Pharmaceuticals B.V., Amsterdam 1083 HN, the Netherlands.; ^2^Department of Pharmacy & Pharmacology, Netherlands Cancer Institute - Antoni van Leeuwenhoek, Amsterdam 1066 CX, the Netherlands.; ^3^SOLVO Biotechnology, Budapest H-1117, Hungary.

**Keywords:** Prostate cancer, docetaxel resistance, cabazitaxel resistance, P-gp, ritonavir

## Abstract

**Aim:** Docetaxel is a microtubule-stabilizing drug used for the treatment of several cancers, including prostate cancer. Resistance to docetaxel can either occur through intrinsic resistance or develop under therapeutic pressure, i.e., acquired resistance. A possible explanation for the occurrence of acquired resistance to docetaxel is increased drug efflux via P-glycoprotein (P-gp) drug transporters.

**Methods:** We have generated docetaxel-resistant cell lines DU-145DOC10 and 22Rv1DOC8 by exposing parental cell lines DU-145DOC and 22Rv1 to increasing levels of docetaxel. Gene expression levels between DU-145DOC10 and 22Rv1DOC8 were compared with those of their respective originator cell lines. Both parental and resistant cell lines were treated with the taxane drugs docetaxel and cabazitaxel in combination with the P-gp/CYP3A4 inhibitor ritonavir and the P-gp inhibitor elacridar.

**Results:** In the docetaxel-resistant cell lines DU-145DOC10 and 22Rv1DOC8, the ABCB1 (P-gp) gene was highly up-regulated. Expression of the P-gp protein was also significantly increased in the docetaxel-resistant cell lines in a Western blotting assay. The addition of ritonavir to docetaxel resulted in a return of the sensitivity to docetaxel in the DU-145DOC10 and 22Rv1DOC8 to a level similar to the sensitivity in the originator cells. We found that these docetaxel-resistant cell lines could also be re-sensitized to cabazitaxel in a similar manner. In a Caco-2 P-gp transporter assay, functional inhibition of P-gp-mediated transport of docetaxel with ritonavir was demonstrated.

**Conclusion:** Our results demonstrate that ritonavir restores sensitivity to both docetaxel and cabazitaxel in docetaxel-resistant cell lines, most likely by inhibiting P-gp-mediated drug efflux.

## INTRODUCTION

Docetaxel is an inhibitor of microtubule depolymerization and an important anticancer agent. In 2004, this drug was approved for the treatment of metastatic castration-resistant prostate cancer (mCRPC)^[1,2]^. The standard of care treatment with docetaxel for mCRPC patients is intravenous administration of 75 mg/m^2^ once every 3 weeks. A common problem with docetaxel treatment is failure attributed to the development of resistance, possibly due to increased efflux from the site of action caused by P-glycoprotein (P-gp)^[3]^. Ritonavir is an HIV protease inhibitor originally used for the treatment of HIV patients, and is now also commonly used as a “booster” of other drugs^[4]^ (most recently in combination with nirmatrelvir for the treatment of COVID-19) due to its ability to inhibit both cytochrome P4503A4 (CYP3A4)^[5]^ and P-gp^[6]^. Targeting P-gp efflux could potentially also be used to reverse docetaxel resistance.

Patients often prefer oral administration of anticancer drugs over intravenous administration^[7]^, as it enables more convenient and flexible dosing schedules. An oral formulation of docetaxel was developed in the form of ModraDoc006 tablets. An important challenge to achieving adequate therapeutic exposure to docetaxel after oral administration is that it is affected by both P-gp-mediated drug efflux, and metabolism and inactivation by the enzyme CYP3A4. Ritonavir inhibits both CYP3A4 and P-gp^[8,9]^ and consequently changes the bioavailability of ModraDoc006, resulting in higher systemic exposure of docetaxel. The combination ModraDoc006/r (“r” denoting co-administration of ritonavir) was tested in the clinic in several studies^[10,11]^. These studies consistently showed an enhanced exposure of docetaxel with no additional toxicity caused by ritonavir. The clinical use of ritonavir as a booster of docetaxel exposure is primarily based on inhibition of CYP3A4. However, in the clinical setting, ritonavir will not only increase the plasma levels of the intended boosted drug docetaxel, but also potentially increase exposure levels of other concomitantly used medications that are CYP3A4 substrates. This is a limitation for using the docetaxel/ritonavir in combination with other CYP3A4 substrate drugs.

In addition, it has been suggested that ritonavir may inhibit intracellular CYP3A4-mediated metabolism in cancer cells. Synergistic anti-proliferative effects of the combination of docetaxel with ritonavir were seen in prostate cancer DU-145 cell cultures and in DU-145 grafted in BNX nu/nu triple immune-deficient mice^[12]^. Furthermore, in a breast cancer model (K14cre;Brca1^F/F^;p53^F/F^ grafted in Cyp3a^-/-^ mice), the efficacy of docetaxel was enhanced when combined with ritonavir^[13,14]^.

Docetaxel is a highly effective mCRPC treatment; however, patients invariably develop resistance to this treatment. Various docetaxel resistance mechanisms in prostate cancer have been described^[15-17]^. In general, the reasons for docetaxel resistance in prostate cancer can be separated into two groups^[18]^. One group is linked to the loss of drug-target engagement, which results in microtubules no longer being stabilized and, thereby, loss of formation of microtubule bundles that normally would lead to cell death. Causes for this effect include tubulin mutations and other tubulin modifications/alterations. Importantly, drug-target interaction is also affected by overexpression of Adenosine triphosphate (ATP) binding cassette transporters, blocking docetaxel from its site of action. The best investigated ATP binding cassette (ABC) transporter is P-gp [or multidrug resistance protein 1 (MDR1)], which is encoded by the multidrug resistance gene ABCB1. The MDR phenotype has been associated with intrinsic resistance to cancer drug treatment, but also has been clearly linked to acquired resistance to a variety of drug classes including anthracyclines, taxanes, and epipodophyllotoxins^[19]^. Increased P-gp expression, in particular, has been associated with docetaxel resistance in prostate cancer^[20]^. Another well-studied group of resistance mechanisms is related to modifications in pathways downstream of target engagement, including: increased BCL2 or MCL1 expression, p53 function loss, up-regulation of Notch, and NF-κB activation. Cabazitaxel, another taxane drug, has a lower affinity for P-gp compared to docetaxel and is registered for the treatment of patients with mCRPC that have progressed during or after docetaxel^[21]^.

Several inhibitors of P-gp were successful in reversal of docetaxel resistance in prostate cancer cell line models. An overview of studies investigating the effect of a variety of drugs in combination with docetaxel aiming to reverse docetaxel resistance in prostate cancer cell assays is shown in Table 1.

**Table 1:** Overview of studies with drugs tested in resistant prostate cancer cell assays

**Authors**	**Docetaxel-resistant cell lines**	**Agents tested**	
O’Neill *et al.*^[22]^	DU-145	Elacridar	P-gp inhibitor
	22Rv1	Elacridar	P-gp inhibitor
	PC3	BAY-11-7082	NF-κB inhibitor
Zhu *et al.*^[23]^	C4-2B	Apigenin	Bioflavonoid
Oprea-Lager *et al.*^[24]^	MLL	MK571	Quinoline derivative
Monteverde *et al.*^[25]^	PC3	Vandetanib	EGFR inhibitor
Zhu *et al.*^[26]^	DU-145	Octreotide	Octapeptide
	PC3	Octreotide	Octapeptide
Zhu *et al.*^[27]^	C4-2B	Bicalutamide	Antiandrogen
	DU-145	Enzalutamide	Androgen-receptor inhibitor
Lin *et al.*^[28]^	DU-145	MP470	AXL inhibitor
	DU-145	R428	AXL inhibitor
	PC3	MP470	AXL inhibitor
	PC3	R428	AXL inhibitor
Luty *et al.*^[29]^	DU-145	Fenofibrate	Fibric acid
	PC3	Fenofibrate	Fibric acid
Wang *et al.*^[30]^	C4-2B	SR2211	RORγ agonist
	DU-145	SR2211	RORγ agonist

P-gp: P-glycoprotein.

Inhibitors of targets other than P-gp restored docetaxel efficacy as well, pointing to additional modes of action of docetaxel resistance beyond P-gp-mediated efflux. Still, most agents studied appear to have a direct or indirect effect on P-gp-mediated drug efflux in prostate cancer cells. Up-regulation of the *ABCB1* gene was identified as a feature in several cell lines resistant to docetaxel, including C4-2B, DU-145, and LNCaP^[31,32]^. Docetaxel resistance in PC3 cell lines seemed independent of P-gp overexpression when other differentially regulated genes were more common^[22,33]^.

The aim of the current study was to generate several docetaxel-resistant prostate cancer cell lines, to investigate the genetic changes that made the cell lines resistant, and to study the potential of ritonavir to reverse resistance to docetaxel and cabazitaxel.

## METHODS

### Materials and reagents

Docetaxel, cabazitaxel, ritonavir, and elacridar were obtained from Selleckchem (Houston, TX, USA), dissolved in DMSO, and stored at -20 °C. From stock solution, target concentrations were made in DMSO. These were then diluted 1:22 in cell culture medium, and of these, 10 µL were taken into 140 µL final cell culture volume. The final DMSO concentration was 0.3% v/v in all experiments. Bacto Agar was from BD Biosciences, CellTiter-Blue® cell viability assay from Promega. Heat-inactivated fetal calf serum (FCS) was from Sigma-Aldrich, gentamicin from Life Technologies, Iscove’s Modified Dulbecco’s Medium (IMDM) and phosphate buffered saline (PBS) Dulbecco’s w/o calcium, magnesium, w/o sodium bicarbonate from Life Technologies, and RPMI 1640-Medium with 25 mM HEPES buffer, with L-glutamine from Biochrom, all from local subsidiaries. Human prostate cancer cell lines were obtained from standard sources: 22Rv1 was from American Type Culture Collection (ATCC, Rockville, MD, USA), whereas DU-145 and PC-3 were obtained from National Cancer Institute (NCI, Bethesda, MD, USA). Protease inhibitors for Western blotting Na_3_VO_4_, NaF, PMSF, and benzonase were obtained from Sigma-Aldrich (Taufkirchen, Germany). The 22Rv1 prostate cancer cell line was obtained from the CWR22R xenograft model. It is an androgen receptor (AR)-positive prostate cancer model. The PC3 cell line, of a low differentiated cell type, was isolated from human prostate cancer bone metastases. It is an androgen-independent prostate cancer cell known to have moderate metastatic potential. The DU-145 cell line was the first prostate cancer cell line and was derived from a brain metastatic prostate tumor. The DU-145 cells do not show AR protein expression.

### Cell culture and generation of docetaxel-resistant prostate cancer sublines

Cells were grown at 37 °C in RPMI 1640 medium supplemented with 10 % (v/v) fetal calf serum and 50 µg/mL gentamicin and passaged twice weekly. The percentage of viable cells was determined by the CASY TT cell counter (OMNI Life Science). DU-145 and 22Rv1 docetaxel-resistant clones were generated by exposure to 2 nM docetaxel and stepwise increase to 4, 6, 8, and finally 10 nM docetaxel. PC-3 cells were exposed to 0.5, 1, 2, and finally 3 nM docetaxel. In brief, after 4 weeks of incubation with docetaxel, cytotoxicity to docetaxel was determined again when sufficient cells were available. Concentrations of docetaxel were increased when the growth of treated cells in the presence of docetaxel was comparable to parental cells. Then, after another 4 weeks, the procedure was repeated. After about 3 months, no further increase in resistance to docetaxel was measured and the cells were stored as aliquots in liquid N_2_. Cells of one aliquot were cultured to have enough sufficient cells (approx. 1-2 weeks). Cells were seeded in the assay plates 24 h before treatment.

### Determination of cytotoxicity

Cytotoxicity was determined in two models: a 2D cell proliferation assay with cells adhering to the tissue plate and a 3D clonogenic assay with cells growing non-attached in soft agar. The viability of cells in the 2D assay was analyzed by using the CellTiter-Blue® Cell Viability Assay. The assay is based on the ability of living cells to convert a redox dye (resazurin) into a fluorescent end-product (resorufin)^[34]^. Briefly, cells were harvested from exponential phase cultures, counted, and plated in 96-well flat-bottom microtiter plates at a cell density of 4,000-20,000 cells/well in a volume of 140 µL per well. After a 24-hour recovery period, 10 μL cell culture medium with or without test compound was added. After 72 h of total incubation, 20 µL/well CellTiter-Blue® reagent was added. After up to four hours, fluorescence (FU) was measured by using an Enspire Multimode Plate Reader. The 3D-assy (clonogenic assay) was carried out in a 96-well plate format using ultralow attachment plates. For each test, cells were prepared as described above and assay plates were prepared as follows: each test well contained a layer of semi-solid medium with tumor cells (50 µL), and 24 h later, a second layer of medium supernatant with or without test compound (100 µL) was added. The cell layer consisted of 2,000 to 7,500 tumor cells per well, which were seeded in 50 µL/well cell culture medium [IMDM, supplemented with 20% (v/v) FCS, 50 µg/mL gentamicin, and 0.4% (w/v) agar]. After 24 h, the soft-agar layer was covered with 100 µL medium without agar, consisting of 90 µL of the same culture and 10 µL of control medium or test compound pre-diluted from DMSO in cell culture medium (see above), and left on the cells for the duration of the experiment (continuous exposure, 100 µL drug overlay). Every plate included six untreated control wells and drug-treated groups. Duplicate values were obtained by testing single values per condition on one plate. Cultures were incubated at 37 °C and 7.5% CO_2_ in a humidified atmosphere for 13 days and monitored closely for colony growth using an inverted microscope. Within this period, tumor growth led to the formation of colonies with a diameter of > 50 µm (area > 2,000 µm^2^). At the time of maximum colony formation, vital colonies were stained for 48 h with a sterile aqueous solution of 2-(4-iodophenyl)-3-(4-nitrophenyl)-5-phenyltetrazolium chloride (INT, 1 mg/mL, 25 µL/well), and colony counts were performed with an automatic image analysis system (CellInsight NXT, Thermo Scientific or Bioreader 5000 V-alpha, BIO-SYS GmbH). In general, results of the 3D clonogenic assay are found to be highly predictive for *in vivo* efficacy of test compounds, including tubulin targeting agents^[35]^.

### Assessment of drug response

Sigmoidal concentration-response curves were fitted through the data points obtained for each tumor model using a 4-parameter non-linear curve fit (Charles River Discovery Research Services Germany Data-Warehouse Software). IC_50_ values are reported as relative IC_50_ values. The relative IC_50_ value is the concentration of the test compound that gives a response (inhibition of colony formation) halfway between the top and bottom plateau of the sigmoidal concentration-response curve (inflection point of the curve). Drug combinations were evaluated using Bliss independence analysis. Measured treated *vs.* control (T/C) values of the combination are shown for each pair of concentrations of the compounds (modeled T/C). The expected effect (= Bliss neutral value) for simple additivity (E12) of a combination was computed by multiplication of the measured effect of compound 1 alone (E1) and effect of compound 2 (E2) alone, with E1 and E2 being fraction unaffected like percentage of viable cells (T/C values) in an inhibition assay as it was used in the present study: Bliss neutral value: E12 = E1 * E2 E12 = T/C [compound 1] * T/C [compound 2]

The difference between the Bliss neutral value and the measured T/C value (= modeled T/C) for the combination was taken: Bliss Index: E12 (Bliss neutral) - T/C [combination]

The results of drug combination tests were presented in heat maps. In the Modeled T/C heat maps, the efficacy of the different combination conditions is shown as the modeled T/C value, which is the mean of the measured T/C values of the individual conditions on a scale ranging from 0 to 1.0, where 1.0 corresponds to a T/C value of 100%. In the Bliss- Modeled T/C, the Bliss index is shown as the difference between the expected T/C value (Bliss neutral) and the measured T/C value (modeled T/C) on a scale ranging from -1.0 to 1.0. Positive values (Bliss Index ≥ 0.15, blue) indicate synergy, negative values (Bliss Index ≤ -0.15, red) indicate antagonism, and zero is the neutral value (white). If the observed effect is consistent across several test concentrations, an additive/synergistic/ antagonistic effect of the combination can be assumed.

### RNA-sequencing

DU-145, DU-145DOC10, 22Rv1, and 22Rv1DOC8 were grown in triplicate and RNA was isolated. Paired-end RNA-sequencing data were generated using the Illumina NovaSeq 6000 with between 60 and 75 k reads per sample. 58,243 targets were tested, of which 16,387 features with a read count equal to zero across all samples were excluded from analysis. Raw expression data were evaluated using four automated outlier tests i.e., sum of Euclidean distance, Hoeffding’s D-statistic, mean Pearson correlation, and the Kolmogorov-Smirnov test statistic. Normalization was carried out using trimmed mean normalization and data were transformed with voom^[36]^. The data were also manually inspected with the density plots, principal component plots, correlation heat map, and principal component analysis association plots. No samples were identified as outliers based on the results of these tests. Two statistical contrasts were performed to determine significantly differentially expressed genes (DEGs) between the docetaxel-resistant and respective parental samples. The difference in cell viability was calculated from independent duplicate/triplicate samples using a Students’ *t*-test. Values *P* < 0.05 were considered statistically significant. Significant differentially expressed genes (DEGs) were identified as those that surpassed a statistical threshold corrected for multiple testing using the false discovery rate (FDR) adjustment < 0.05 and a ≥ 2-fold change in expression value.

### Western blotting

Cells were grown in cell culture flasks, washed with PBS twice, and then detached by incubation in PBS with 5% EDTA at 37 °C for 5 min. Cells were washed with PBS, centrifuged down at 300 g for 5 min, transferred into 1.5 mL Eppendorf tubes, and centrifuged again. The resulting pellet was snap-frozen in liquid nitrogen. On the day of lysis, the pellet was re-suspended in 300 µL of pre-cooled OT-lysis buffer (20 mM Tris pH 7.4 with 100 mM NaCl, 1 mm EDTA, 1 mM EGTA, 1% NP-40, 0.5% deoxycholate, 10 mM NaP_2_O_7_ and with the protease inhibitors Na_3_VO_4_, NaF, PMSF, and benzonase, all from Sigma-Aldrich, Taufkirchen, Germany). The cells were lysed by incubation on ice for 15 min. After 5, 10, and 15 min, the tube was pulse-vortexed for 3 s. Samples were then centrifuged at 16,000 g, 4 °C for 20 min. The supernatants of about 200 µL were transferred into new tubes and an aliquot was taken for protein concentration determination. Samples were snap frozen until further use. Protein concentrations were measured using the DC Protein Assay Kit II (BioRad, #500-0112). The samples were thawed on the day of western blotting and their concentration was adjusted to 2.5 mg/mL. Lysates were denatured using 2× Laemmli Sample Buffer (Biorad, #1610737) with TCEP (Sigma, #646547) as the reducing agent. The mixture was incubated for 5 min at 95 °C, while shaking at 600 rpm in a Thermomixer comfort (Eppendorf, #5360 000.011). The denatured lysates were spun down in a tabletop centrifuge and 15 µL lysate was loaded into a Criterion TGX precast gradient gel (Biorad, #5671095). Electrophoresis was performed for 50 min at 180 V, using a peqPower300 power supply (PeqLab, #55-E300-230V) in a Criterion Cell chamber (Biorad, #165-6001). The transfer was done using a Trans-Blot Turbo Transfer System (Biorad, #170-4155) and a ready-to-use PVDF membrane (Biorad, #170-4157) for 10 min at 2.5 A. The membrane was blocked in 5% (w/v) milk (Roth, #T145.3) in TBS/T (Sigma, #T5912 diluted 10-fold and Sigma, #P1379, diluted 1000-fold). The primary rabbit monoclonal IgG antibodies were diluted in TBS/T with 5% (w/v) BSA (Sigma, #A2153) and incubated for MDR1 shaking at 4 °C overnight and for β-actin 1hr at RT. The secondary goat anti-rabbit IgG antibody was diluted in TBS/T with 5% (w/v) milk and shaken with the membrane for 1 h at RT. Detection was performed using the Amersham ECL Prime reagent (GE Healthcare, #28980926) in an Image Quant 4000 (Cytiva, #28955810).

### Caco-2 substrate assessment of docetaxel and bidirectional permeability assays

Bidirectional transport of docetaxel was determined through Caco-2 cell monolayers. Assay buffer containing docetaxel at 5 and 10 μM with and without reference inhibitor (10 μM valspodar or 50 μM ritonavir) was added to the appropriate apical or basolateral chamber. Samples were taken from the helper plate before the incubation and from the donor chambers after the incubation to determine the initial concentration (C0) and recovery (R) of docetaxel and the controls. After incubation, the receiver chambers were sampled to determine the amount of translocated docetaxel and controls. Samples were analyzed using LC-MS/MS. E3S (0.2 μM) for BCRP and digoxin (5 μM) for MDR1 were used as positive control substrates, while Ko143 (5 μM) for BCRP and valspodar (10 μM) for MDR1 were applied as reference inhibitors.

Bidirectional permeability assays were performed using Caco-2 (C2Bbe1 clone, ATCC-2102) cell monolayers. The cells were cultured in Dulbecco’s Modified Eagle’s Medium with 4.5 g/L glucose (DMEM) supplemented with 10% (v/v) fetal bovine serum (FBS) at 37 °C in a humidified atmosphere containing 5% CO_2_ in cell culture flasks prior to seeding into collagen-coated 24-transwell inserts. Cells were cultured on the inserts with 400 μL medium per well on the apical side and 25 mL in a single-well receiver tray for all 24 wells on the basolateral side for 20-23 days. The medium was changed 24 h before the experiment. In all experiments, cells were pre-incubated in assay buffer for 15 min to allow cells to adjust to the assay conditions. Permeability incubations were carried out in Hank’s Balanced Salt Solution (HBSS) at 37 °C. The apical-to-basolateral permeability of Lucifer Yellow (LY) was assessed as a low permeability control. LY was also incubated in the presence of 5 and 10 μM docetaxel to assess the effect of the compound on the monolayer integrity. LY samples were analyzed by measuring fluorescence with the following wavelengths: excitation - 430 nm, emission - 520 nm. Estrone-3-sulfate (E3S) and digoxin efflux ratios (ERs) were determined as positive controls for BCRP and MDR1 function, respectively. The transepithelial electric resistance (TEER) of each well was s of data over distance d can be represented asat 5 and 10 μM in the absence and presence of valspodar (10 μM) or ritonavir (50 μM) was then added to the appropriate apical (400 μL) or basolateral chambers (800 μL). After a 60-minute incubation at 37 °C, aliquots (100 μL) were taken from the receiver chambers to determine the amount of translocated docetaxel and controls. Samples were taken from the donor chambers before and after a 120-minute incubation to determine the initial concentrations (C0) and recovery (R) of the test compounds and the controls. The cellular compartment was also sampled for a more complete recovery of docetaxel. Bidirectional transport of docetaxel was determined by Liquid chromatography-Mass Spectrometry (LC-MS). The bioanalysis of docetaxel was performed with the . A minimum of six calibration concentration levels was used for fitting the calibration curve.

The following equation was used to calculate apparent permeability coefficient (Papp):

**Figure:**
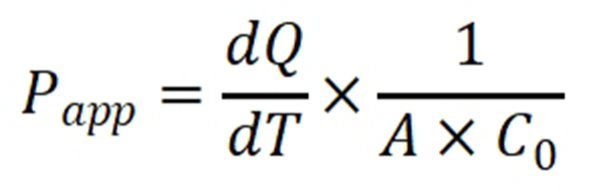


*dQ*: amount of transporter test drug in pmol; *dT*: incubation time in seconds; *A*: surface of porous membrane in cm^2^ (standard: 0.7); *C_0_*: initial concentration of test agent in the donor chamber in pmol/cm^3^.

The efflux ratio (ER) is given as:

**Figure:**
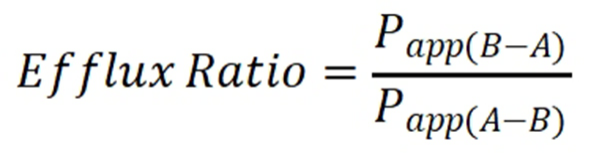


*P_app_*_(_ *_A-B_*_)_: apparent permeability in the apical to basolateral direction; *P_app_*_(_ *_B-A_*_)_: apparent permeability in the basolateral to apical direction.

Recovery (R) was calculated according to the following formula to allow for estimation of metabolism and/or non-specific binding:

**Figure:**
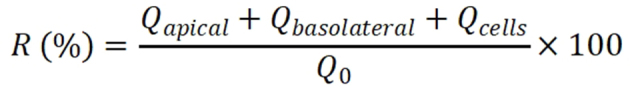


*Q_apical_*: quantity of TA detected on apical side in pmol; *Q_basolateral_*: quantity of TA detected on basolateral side in pmol; *Q_cells_*: quantity of TA in the cellular compartment in pmol; *Q_0_*: quantity of TA detected at t_0_ in pmol.

Assay parameters and treatment groups are summarized in Table 2.

**Table 2:** Assay parameters for bidirectional permeability assessment in Caco-2 cells

**Compound**	**Direction**	**Concentration**	**Incubation time (min)**
Docetaxel	A-B/B-A	5 and 10 µM	60 and 120
Docetaxel + valspodar	A-B/B-A	5 and 10 µM + 10 µM	60 and 120
Docetaxel + ritonavir	A-B/B-A	5 and 10 µM + 50 µM	60 and 120
LY	A-B	40 µg/mL	120
LY + docetaxel (TA applied apically)	A-B	40 µg/mL + 5 and 10 µM	120
LY + docetaxel(TA applied basolaterally)	A-B	40 µg/mL + 5 and 10 µM	120
Digoxin	A-B/B-A	5 µM	60
Digoxin + valspodar	A-B/B-A	5 µM + 10 µM	60
E3S	A-B/B-A	0.2 µM	60
E3S + Ko143	A-B/B-A	0.2 µM + 5 µM	60

Permeability controls LY: Lucifer Yellow. Functional controls: digoxin and E3S. Digoxin control confirmed the functional activity of P-gp, while the monolayer integrity marker indicated that the applied docetaxel concentrations did not harm the Caco-2 cells (data not shown). A-B: Apical to basolateral direction; B-A: basolateral to apical direction; TA: test agent; E3S: estrone-3-sulfate; P-gp: P-glycoprotein.

## RESULTS

### Activity of docetaxel and ritonavir in a cell line panel

Before starting experiments in resistant cell lines, the activity of docetaxel, ritonavir, and the combination of both drugs was established in a panel of 15 tumor cell lines, including four prostate cancer cell lines (22Rv1, DU-145, PC-3, and PC-3M) [Table 3].

**Table 3:** IC_50_ values of docetaxel, ritonavir, and the combination of both drugs in 15 tumor cell lines

**Tumor/cell line**	**Site of origin**	**Source**	**Relative docetaxel IC_50_ (µM)**	**Relative ritonavir IC_50_ (µM)**	**Docetaxel + ritonavir**
GXA 3011	Stomach	PDX-derived, CRL	0.0005	> 30.0000	Additive
GXA NUGC-4	Lymph node	JCRB	0.0055	1.2716	Additive
LXFA 737	Lung	PDX-derived, CRL	0.0022	> 30.0000	Additive
LXFA EKVX	Lung	NCI	0.0019	> 30.0000	Additive
MAXFLB MCF7	Not known	NCI	0.0011	> 30.0000	Additive
MAXFTN CAL-51	Not known	DSMZ	0.0017	> 30.0000	Additive
MAXFTN MCF 10A	Breast	ATCC	0.0007	> 30.0000	Synergy
MAXFTN MX1	Not known	NCI	0.0019	> 22.6260	Additive
OVXF 899	Ovary	PDX-derived, CRL	0.0202	25.6648	Synergy
OVXF EFO-27	Not known	DSMZ	0.0007	> 30.0000	Additive
OVXF NCI-ADR-RES	Not known	NCI	> 0.1000	> 30.0000	Synergy
PRXF 22Rv1	Prostate	ATCC	0.0015	29.3303	Additive
PRXF DU-145	Prostate	NCI	0.0009	16.2600	Slight antagonism
PC-3	Prostate	NCI	0.0019	21.7296	Additive
PC-3M	Prostate	NCI	0.0024	23.3127	Additive

JCRB: Japanese Collection of Research Bioresources Cell Bank; NCI: National Cancer Institute; DSMZ: German Collection of Microorganisms and Cell Cultures; ATCC: American Type Culture Collection.

Docetaxel was tested as a single agent at 10 concentrations in the 2D proliferation assay to identify an appropriate test range for subsequent combination experiments. Ritonavir tested as a single agent showed no considerable or only low activity at the concentration ranges tested. Docetaxel inhibited tumor cell growth in a concentration-dependent manner with mean relative IC_50_ values of 2.6 nM. For drug combination tests, the docetaxel concentrations were selected between 0.1 and 100 nM, whereas the ritonavir concentrations used for all 15 cell lines were 0.3, 0.949, 3, 9.49 and 30 µM. Docetaxel and ritonavir combinations were tested using a 5 × 5 matrix layout 2D proliferation assay and Bliss independence analysis was performed. The combination of docetaxel and ritonavir was synergistic in ovarian cancer cell lines 899 and NCI-ADR-RES as well as for the triple-negative breast cancer cell line MCF 10A. Ritonavir induced an increase in sensitivity to docetaxel in these cell lines. In all other cell lines, except the prostate cancer cell line DU-145 where a tendency to slight antagonism was observed, docetaxel and ritonavir had limited additive effects. An overview of all drug combination test results is provided in Table 4.

**Table 4:** Docetaxel in combination with ritonavir was investigated in 15 tumor cell lines

**Cell Line**	Ritonavir + docetaxel^*^	ABCB1 expression^**^	CYP3A4 expression^**^
GXA 3011	Additive	2.3	2.4
GXA NUGC-4	Additive	8.2	6.6
LXFA 737	Additive	2.3	2.3
LXFA EKVX	Additive	6.1	2.6
MAXFLB MCF-7	Additive	2.3	2.3
MAXFTN CAL-51	Additive	5.7	2.3
MAXFTN MCF 10A	Synergy	2.3	2.3
MAXFTN MX1	Additive	2.2	2.3
OVXF 899	Synergy	8.3	3.1
OVXF EFO-27	Additive	5.1	2.3
OVXF NCI-ADR-RES	Synergy	n.a.^*^	n.a.
PRXF 22RV1	Additive	2.3	2.4
PRXF DU-145	Slight antagonism	2.3	2.4
PRXF PC-3	Additive	2.3	2.4
PRXF PC-3M	Additive	2.3	2.4

^*^Synergy: BI ≥ 0.15 in > 3/25 conditions, slight synergy: BI ≥ 0.15 in 3/25 conditions, antagonism: BI ≤ -0.15 in > 3/25, conditions, slight antagonism: BI ≤ -0.15 in 3/25 conditions, additive effect: -0.15 < BI < 0.15 in > 22/25 conditions. ^**^Analysis of gene expression levels was done using Affymetrix Human Genome U133 Plus 2 Array platform. Classification of expression levels is based on whole compendium data set. conditions, slight antagonism: BI ≤ -0.15 in 3/25 conditions, additive effect: -0.15 < BI < 0.15 in > 22/25 conditions. ^***^Analysis of gene expression levels was done using Affymetrix Human Genome U133 Plus 2 Array platform. Classification of expression levels is based on whole compendium data set. For OVXF NCI-ADR-RES, no compendium data concerning ABCB1 expression are available, but it has been described as having a high expression level of P-gp. P-gp: P-glycoprotein.

### Generation of docetaxel-resistant clones

**Figure 1:**
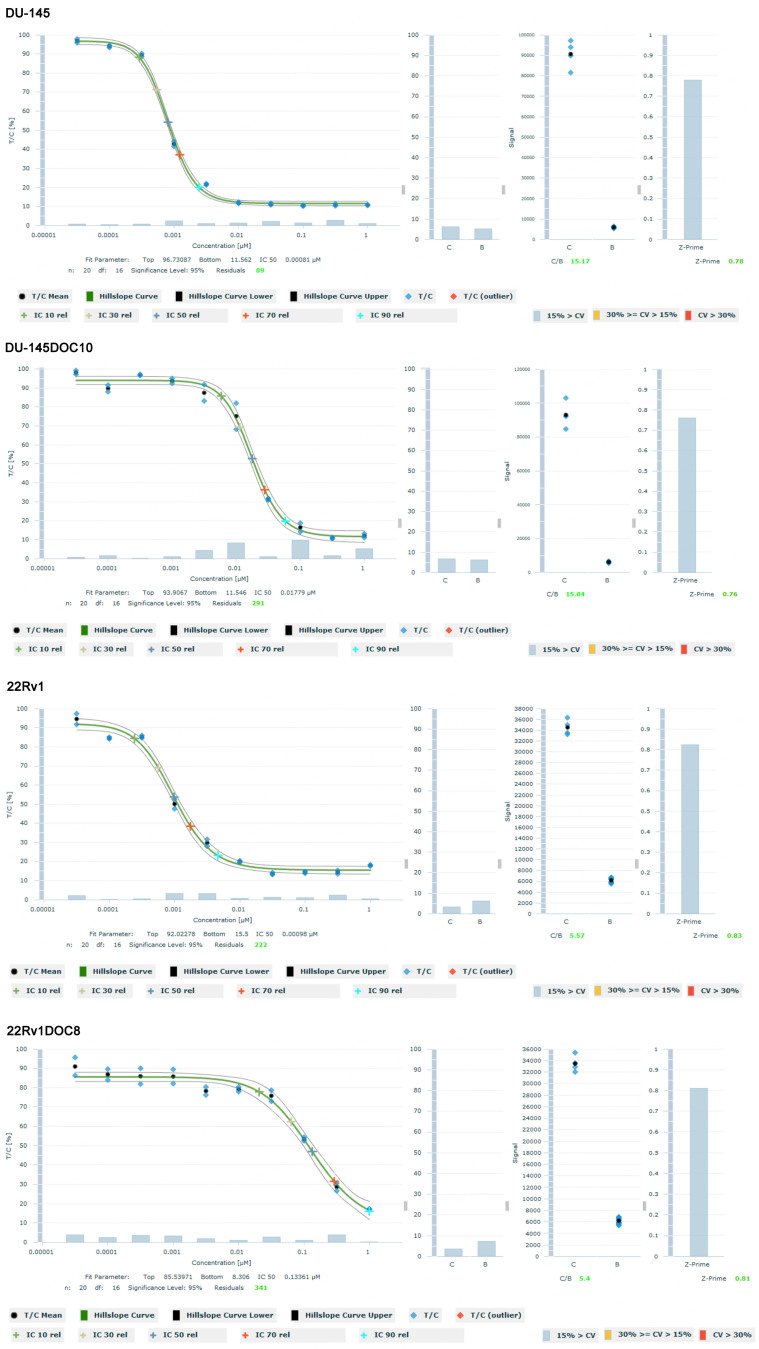
Docetaxel concentration-effect curves in cell lines in 2D culture. The prostate cancer cell lines 22Rv1 and DU-145 were exposed to increasing concentrations of docetaxel over a period of 3 months. Clones of the parental cell lines DU-145 became docetaxel-resistant for 8 and 10 nM (DU-145DOC10). The cell line 22Rv1 became docetaxel-resistant for 4, 6, and 8 nM (22Rv1DOC8).

The DU-145 and 22Rv1 resistant sublines were generated by exposure to 2 nM docetaxel and stepwise increase to 4, 6, 8, and finally 10 nM docetaxel. PC-3 cells were exposed to 0.5, 1, 2, and finally 3 nM docetaxel. This resulted in increased resistance to docetaxel in DU-145 and 22Rv1 [Figure 1], but only imperceptibly for the PC-3 assay. Due to the lack of increased resistance for docetaxel in the PC-3 cells, these cells were not used for further experiments. Inhibitory concentrations of docetaxel in parental and docetaxel-resistant DU-145, 22Rv1, and PC-3 cells have been listed in Table 5.

**Table 5:** Docetaxel relative IC_50_ values as determined in the 2D proliferation assay for parental and resistant cell lines

**Cell line**	**Docetaxel passage concentration**	**Docetaxel IC_50_ (nM)**	**Relative resistance**
DU-145		0.8	
DU-145DOC10^*^	10 nM	17.8	22.3
22Rv1		1.0	
22Rv1-DOC8^*^	8 nM	133.6	133.6
PC-3		1.1	
PC-3DOC3^*^	2 nM	4.4	4.0

Determined in the 2D proliferation assay. ^*^Resistant cell lines.

### Characterization of parental and resistant clones

**Figure 2:**
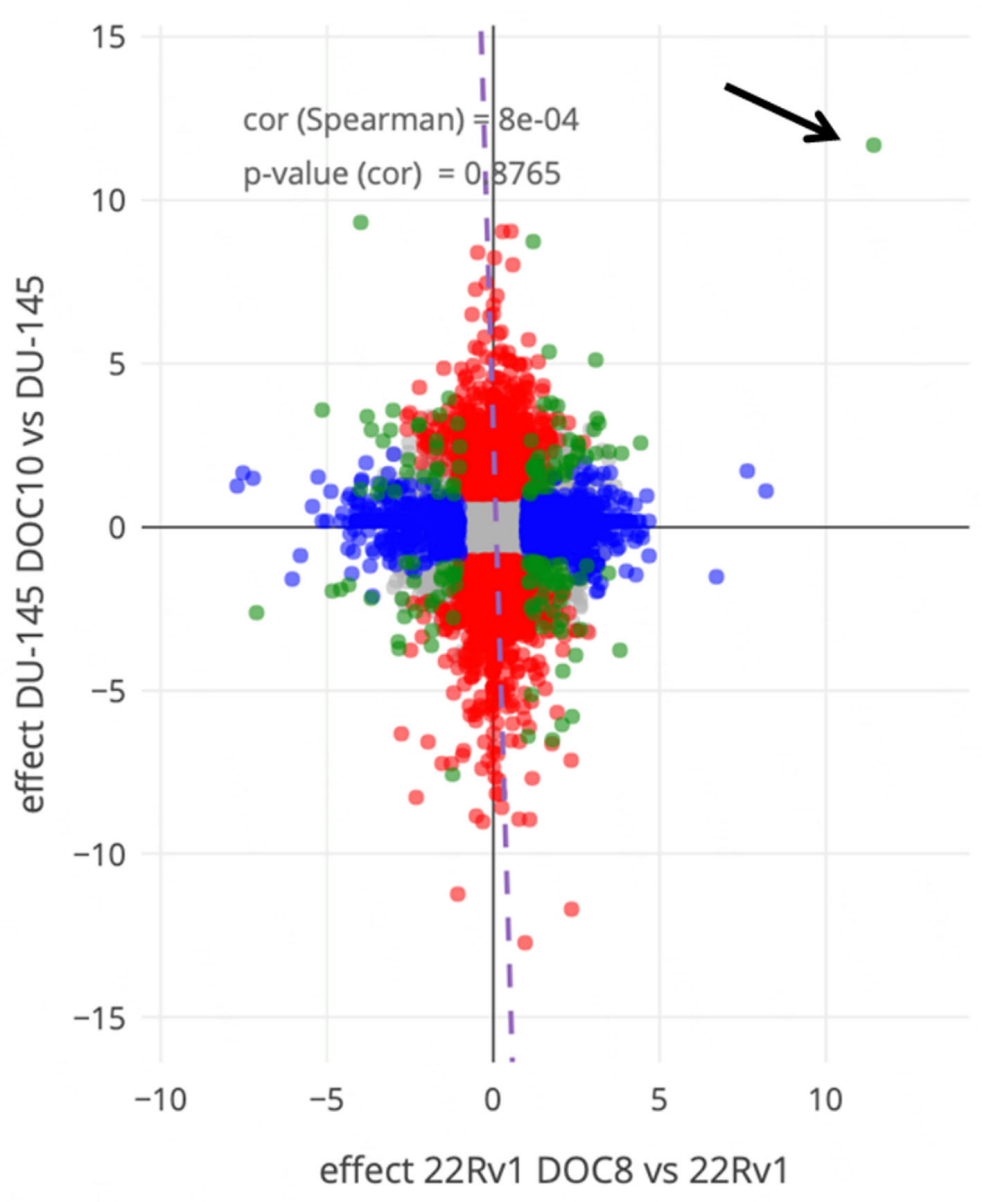
Double volcano plot of differential gene expression between the parental human prostate cancer cell lines DU-145 and 22Rv1 and their docetaxel resistance generated clones DU-145DOC10 and 22Rv1DOC8. Red dots are differentially expressed genes between DU-145 *vs.* DU-145DOC10, blue for 22Rv1 *vs.* its resistant clone 22Rv1DOC8. Green dots are differentially expressed genes in both setups. The grey area represents not significantly regulated genes. Cut-offs of 2-fold and a FDR *P* < 0.05 were used. Note the extreme differentially and significantly expressed green dot for MDR1 (arrow). FDR: False detection rate; MDR1: multidrug resistance protein 1.

To determine the mechanism of resistance to docetaxel in the different cell lines, genome-wide screening using RNA-seq was applied. RNA expression levels of docetaxel-resistant cell lines DU-145DOC10 and 22Rv1DOC8, as well as their parent cell lines DU-145 and 22Rv1, were matched. Of each cell line, RNA was isolated from three different cultures (triplicates). 58,243 features were generated, of which 16,387 features with reads equal to zero were eliminated. After rigorous quality control (see methods), no outliers were detected, and the data were evaluated. 2,360 DEGs were identified between DU-145 DOC10 and DU-145 samples, and 1,058 DEGs were identified between 22Rv1 DOC8 and 22Rv1 [Figure 2].

The genes that were most highly up-regulated in DU-145DOC10 samples compared to DU-145 were those associated with metabolism and homeostasis, including ABCB1 (3287-fold), Dermatan Sulfate Epimerase Like (DSEL, 530-fold), Glypican 4 (GPC4, 260-fold), Matrix Metall-Protease 1 (MMP1, 60-fold), Intern Subunit alpha 4 (ITGA4, 45-fold), and Glypican 6 (GPC6, 44-fold). The most down-regulated genes in DU-145DOC10 samples compared to DU-145 were genes associated with signaling pathways, such as Butyrylcholinesterase (BCHE, 6770-fold), Secreted Frizzled Related Protein (SFRP1, 3325-fold), PBX Homeboy 1 (521-fold), R-Spondin (RSOP3, 497-fold), and Glucagon Like Peptide 2 Receptor (GLP2R, 163-fold), among others. Some CYP4F-family members were down-regulated, but CYP3A4 was 1.7-fold up-regulated. In the 22Rv1DOC8 clone *vs.* the 22Rv1 cell line, genes associated with metabolic or homeostasis pathways were most up-regulated, including ABCB1 (2772-fold), Glutathione S Transpherase Pi1 (GSTP1, 20-fold), GATA5 (19-fold), SERPINA4 (14-fold). The most down-regulated genes in 22Rv1 DOC8 samples compared to 22RV1 were genes associated with neuronal system pathways, such as KCNJ8 (208-fold), SLITRK2 (150-fold), GABA type A receptor (GABRA2, 56-fold), and ABCC9 (30-fold), among others. Some CYP4F-family members were also down-regulated as with DU-145DOC10, but here CYP3A4 was 4-fold down-regulated. It was evident that, in both docetaxel-resistant cell lines, the gene that was up-regulated the most and with the highest significance was ABCB1, 3287-fold in DU-145 DOC10 and 2773-fold in 22Rv1DOC8.

**Figure 3:**
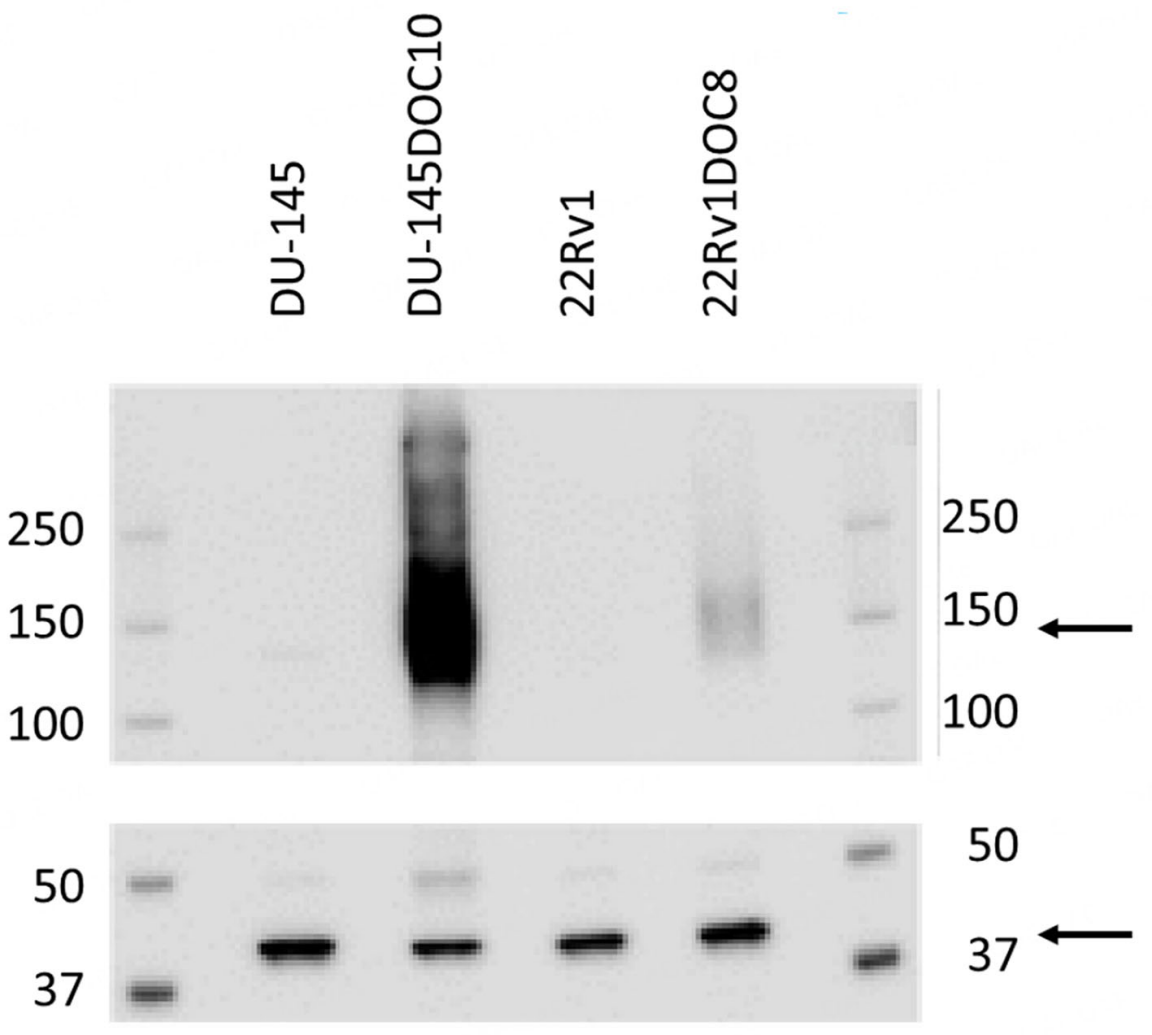
Western blot for the P-gp protein in the parental human prostate cancer cell lines (DU-145 and 22Rv1) and their docetaxel resistance clones (DU-145DOC10 and 22Rv1DOC8). The size of the P-gp protein is indicated by an arrow (130-180 kDa). The lower panel shows equal load by β-actin staining (45 kDa, arrow). P-gp: P-glycoprotein.

The gene expression data for ABCB1 were then confirmed by western blotting. Both parental cell lines DU-145 and 22Rv1 expressed no detectable amount of the P-gp protein. In both docetaxel- resistant cell clones DU-145DOC10 and 22Rv1DOC8, P-gp expression increased, but was especially high in DU-145DOC10 [Figure 3].

### Reversal of resistance to docetaxel

**Figure 4:**
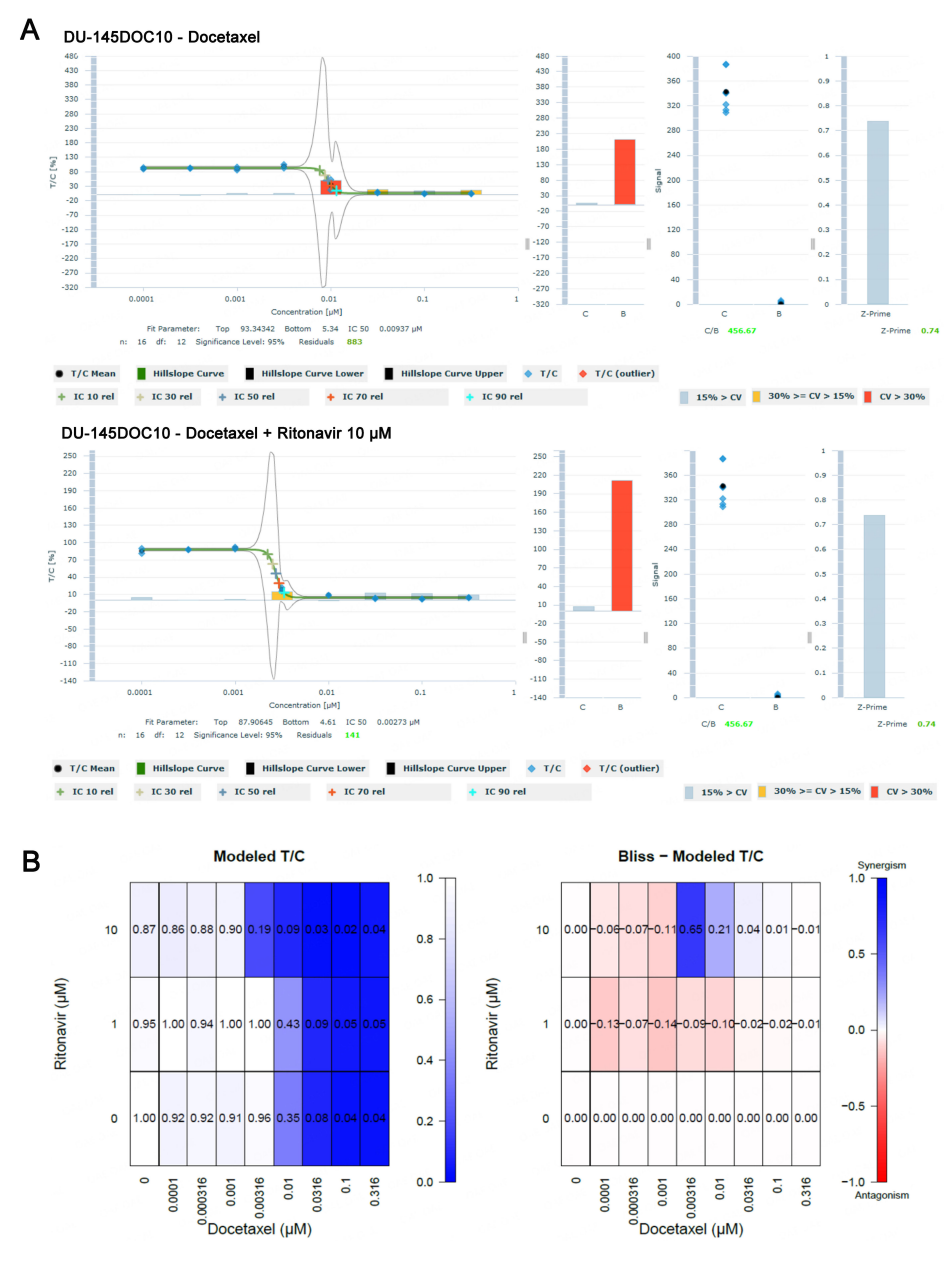
Effect of the docetaxel ritonavir combination in the docetaxel-resistant DU-145DOC10 cell line in the 3D clonogenic assay. (A) Concentration-effect curves showing a decrease of the docetaxel IC_50_ from 9.5 to 2.8 nM in the presence of 10 μM ritonavir; (B) Bliss Modeled T/C analysis shows synergistic effects at 10 μM ritonavir when combined with medium concentrations of docetaxel (3.16 and 10 nM).

DU-145 and docetaxel-resistant DU-145DOC10 cell lines were treated with docetaxel in combination with ritonavir in an 8 × 2 matrix combination setup using the 3D tumor clonogenic assay. The baseline IC_50_ value for docetaxel (i.e., without ritonavir) in the DU-145 cell line was established at 2.9 nM and in the resistant DU-145DOC10 at 9.5 nM; a relative resistance of 3.3. The addition of 10 µM ritonavir to docetaxel in the DU-145DOC10 cell line increased the sensitivity to docetaxel, resulting in a 3.4-fold lower IC_50_ value of 2.8 nM, which is almost identical to the IC_50_ value of the parental cell line. Bliss independence analysis of the DU-145 DOC10 assay showed synergistic effects at 10 μM ritonavir when combined with medium concentrations of docetaxel (3.16 and 10 nM). This confirmed the back-shift of the concentration-effect curves and indicates that the addition of 10 μM ritonavir reverses the resistance to docetaxel. The concentration-effect curve of the docetaxel/ritonavir combination, as well as the Bliss- Model T/C heat map of the DU-145DOC10 assay [Figure 4].

**Figure 5:**
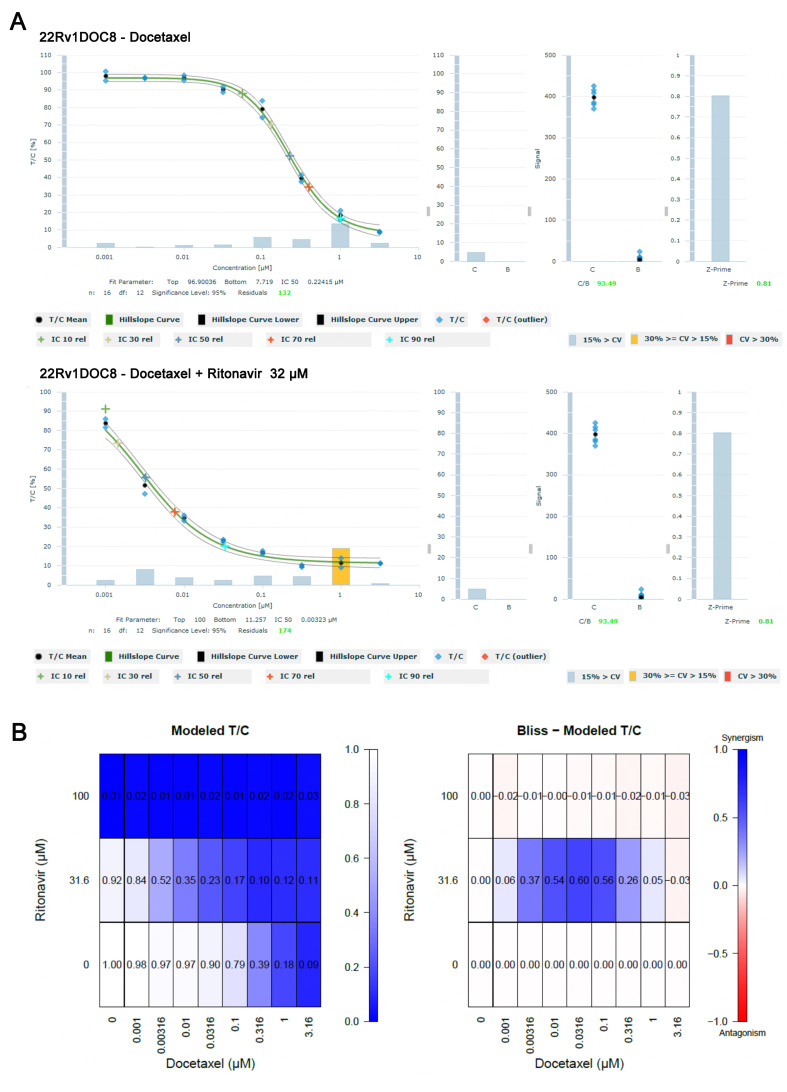
Effect of the docetaxel ritonavir combination in the docetaxel-resistant 22Rv1DOC8 cell line in the 3D clonogenic assay. (A) Concentration-effect curve showing a decrease of the docetaxel IC_50_ from 219 to 26 nM in the presence of 32 μM ritonavir; (B) Bliss Modeled T/C analysis shows synergistic effects at 32 μM ritonavir when combined with medium concentrations of docetaxel (3.16 and 10 nM).

Similarly, parental 22Rv1 and docetaxel-resistant 22Rv1DOC8 cells were treated with docetaxel in combination with ritonavir in an 8 × 2 matrix combination setup using a 3D tumor clonogenic assay. Bliss independence analysis was used to assess synergistic effects. The baseline IC_50_ value for docetaxel in the 22Rv1 and 22Rv1DOC8 cell lines was established at 1.3 and 54.6 nM, respectively, i.e., a relative resistance of 42.0. The addition of 10 µM ritonavir to docetaxel in the 22Rv1DOC8 cell line reduced the IC_50_ value to 46.2, which is still a 36-fold higher relative resistance as compared to the IC_50_ value of 1.3 in the parental cell line. At a concentration of 32 μM, ritonavir decreased the relative IC_50_ value of docetaxel from 219 to 26 nM (84-fold) in the 22Rv1DOC8 cell line and strong synergistic effects were observed at mid concentrations of docetaxel (3.16 to 316 nM) as shown in shown in Figure 5.

**Figure 6:**
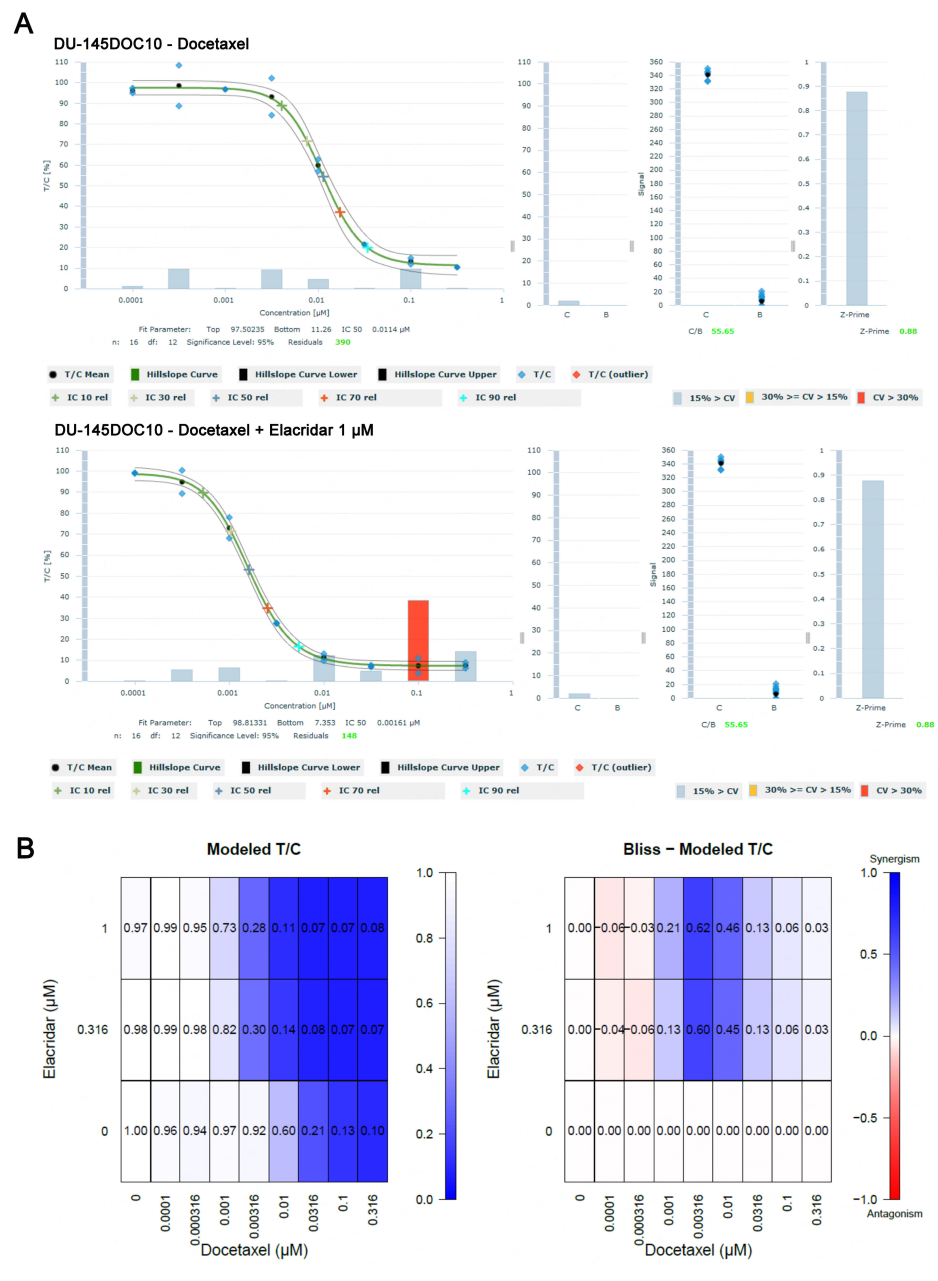
Effect of the docetaxel-elacridar combination in the docetaxel-resistant DU-145DOC10 cell line in the 3D clonogenic assay. (A) Concentration-effect curve showing a decrease of the docetaxel IC_50_ from 11.4 to 1.9 nM combined with 1 μM elacridar and from 11.4 to 1.6 nM combined with 1 μM elacridar; (B) Bliss Modeled T/C analysis shows synergistic effects at both test concentrations of elacridar (0.316 and 1 µM) when combined with medium concentrations of docetaxel (3.16 and 10 nM).

To further investigate if ABCB1 up-regulation affects docetaxel-induced resistance, we used elacridar, a specific P-gp inhibitor, and compared the IC_50_ values of docetaxel in parental and docetaxel-resistant cell lines. In this confirmatory experiment, the objective was to investigate the ability of elacridar to revert resistance towards docetaxel in 22Rv1DOC8 and DU-145DOC10. For this purpose, resistant and parental cell lines were treated with docetaxel in combination with elacridar using the 3D clonogenic experimental setup. Bliss independence analysis was used to assess synergistic effects. The IC_50_ value for docetaxel in the DU-145 cell lines was established at 1.1 nM (DU-145) and 11.7 nM (DU-145-DOC10) in this experiment. For the 22Rv1 cell lines, the docetaxel IC_50_ value was 1.3 (22Rv1) and 134 (22Rv1DOC8). In the resistant cell lines, the relative IC_50_ value of docetaxel decreased 6- and 7.1-fold in DU-145DOC10 and 91- and 116-fold in 22Rv1DOC8 in the presence of 1 or 10 µM elacridar. The elacridar concentrations of 0.316 and 1 µM with docetaxel showed synergistic effects in the DU-145DOC10 assay in the mid concentrations of docetaxel (3.16 and 10 nM). The concentration-effect curve of the docetaxel/elacridar combination, as well as the Bliss Model T/C heat map of the DU-145DOC10 assay [Figure 6].

The 0.316 and 1 µM elacridar concentrations showed strong synergistic effects with docetaxel in the 22Rv1DOC8 assay almost throughout the whole concentration range of docetaxel (1 to 316 nM). Thus, the resistance-reversing effect of ritonavir that was observed in the previous experiment combining docetaxel with ritonavir could be reproduced by elacridar in both DU-145DOC10 and 22Rv1DOC8 cell lines, strongly suggesting that inhibition of P-gp is responsible for this effect. Elacridar alone had no effect on the parental cells (data not shown). Ritonavir itself was also not toxic in the used concentrations (data not shown). Results on the reversal of resistance by ritonavir and elacridar in both cell lines have been summarized in Table 6.

**Table 6:** Docetaxel IC_50_ values combined with ritonavir or elacridar for DU-145 and 22Rv1 cell lines *vs.* DU-145 DOC10 and 22Rv1 DOC8 cell lines

	**Docetaxel IC_50_ (nM)**		**Relative resistance**
**Ritonavir (µM)**			
	**DU-145**	DU-145DOC10^*^	
-	2.9	9.5	3.3
10	1.7	2.8	1.0
	**22Rv1**	22Rv1DOC8^*^	
-	1.3	54.6	42.0
10	1.8	46.2	35.5
32		3.0	2.3
**Elacridar (µM)**			
	**DU-145**	DU-145DOC10^*^	
-	1.1	11.7	10.6
1		1.6	1.5
	**22Rv1**	22Rv1DOC8^*^	
-	1.3	134.0	103.1
1		1.3	1.0

Determined in the 3D clonogenic assay. ^*^Resistant cell lines.

### Reversal of resistance to cabazitaxel

The docetaxel-resistant prostate cancer sublines were tested for sensitivity to cabazitaxel, an analog compound of docetaxel, to investigate whether ritonavir could also reverse resistance to this second taxane. DU-145 DOC10 and 22Rv1 DOC8 and their respective parental cell lines were treated with cabazitaxel in combination with ritonavir in an 8 × 2 matrix combination setup using a 3D tumor clonogenic assay. Docetaxel-resistant DU-145DOC10 and 22Rv1DOC8 sublines were also resistant to cabazitaxel. DU-145DOC10 cells were 2.5 times less sensitive to cabazitaxel than the parent DU-145 cells (IC_50_ of 1.5 nM in DU-145DOC10 *vs.* an IC_50_ of 0.6 nM in DU-145). 22Rv1DOC8 cells were 13.6 times less sensitive to cabazitaxel than the parent 22Rv1 cells (IC_50_ of 9.5 nM in 22Rv1DOC8 *vs.* an IC_50_ of 0.7 nM in 22Rv1). Similar to the observations with the docetaxel ritonavir combination, cytotoxicity to cabazitaxel was restored with 10 µM ritonavir in DU-145DOC10 (IC_50_ of 0.6 nM and a relative resistance of 1.0) and with 32 µM ritonavir in 22Rv1DOC8 (IC_50_ of 0.6 nM and a relative resistance of 1.0). These results have been summarized in Table 7.

**Table 7:** Cabazitaxel IC_50_ values combined with ritonavir for parental cell lines *vs.* the docetaxel-resistant cell lines

**Ritonavir (µM)**	**Cabazitaxel IC_50_ (nM)**		**Relative resistance**
	**DU-145**	DU-145DOC10^*^	
-	0.6	1.5	2.5
10	0.3	0.6	1.0
	**22Rv1**	22Rv1DOC8^*^	
-	0.7	9.5	13.6
10	0.7	5.2	7.4
32	0.7	0.7	1.0

Determined in the 3D clonogenic assay. ^*^Resistant cell lines.

**Figure 7:**
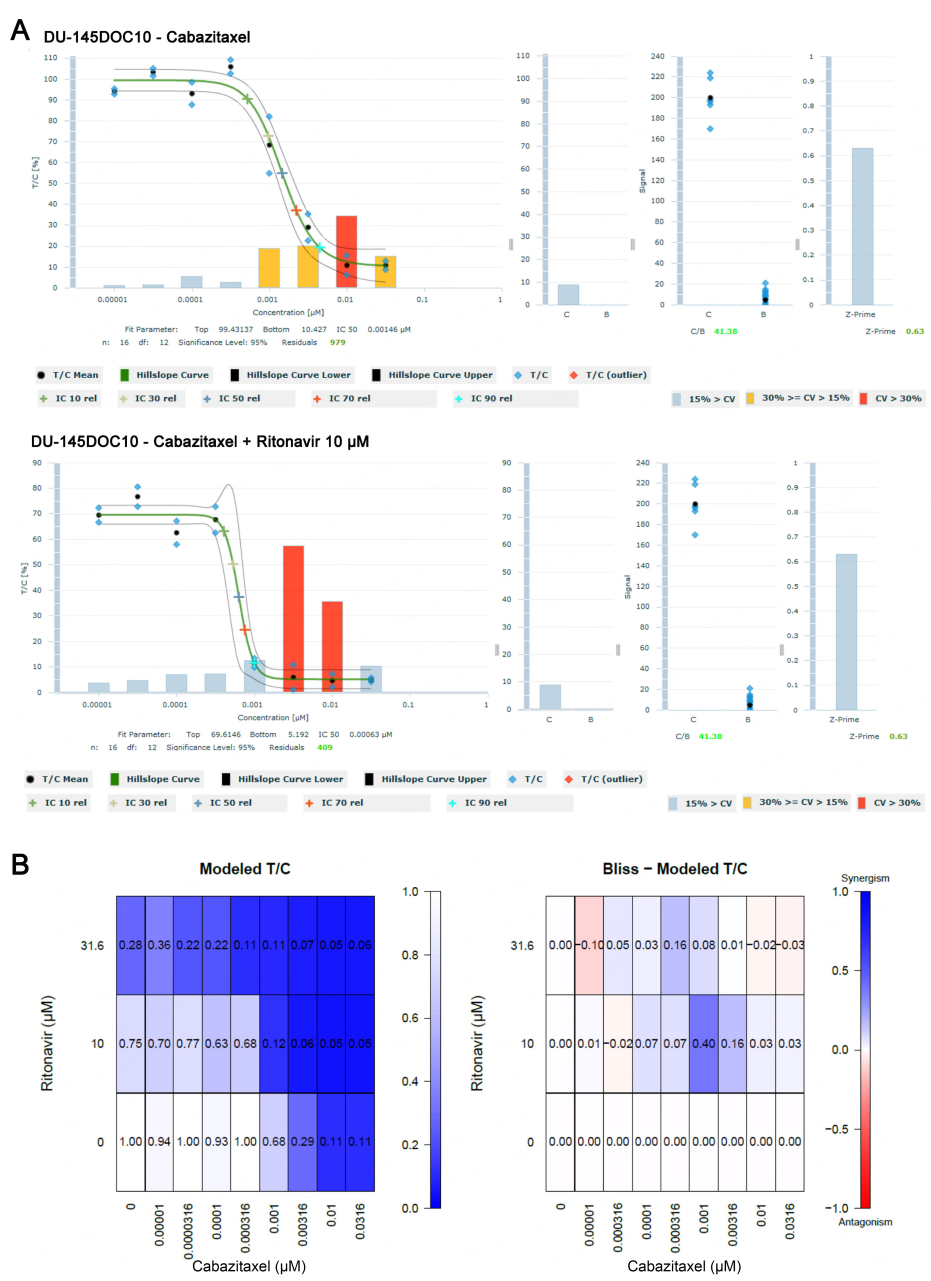
Effect of the cabazitaxel ritonavir combination in the docetaxel-resistant DU-145DOC10 cell line in the 3D clonogenic assay. (A) Concentration-effect curve showing a decrease in the cabazitaxel IC_50_ from 1.5 to 0.6 nM in the presence of 10 μM ritonavir; (B) Bliss Modeled T/C analysis shows synergistic effects at 10 μM ritonavir when combined with cabazitaxel concentrations of 1 and 3.16 nM.

In the Bliss analysis of the DU-145DOC10 assay, synergistic effects were observed of 10 µM ritonavir combined with 1 or 3.16 nM cabazitaxel. The concentration-effect curve of the cabazitaxel/ritonavir combination, as well as the Bliss Model T/C heat map of the DU-145DOC10 assay [Figure 7]. In the 22Rv1DOC8 assay, synergistic effects were observed in the combination of 32 µM ritonavir and 1 to 10 nM cabazitaxel.

### Interaction of docetaxel with the P-gp transporter

The human Caco-2 gastrointestinal cell line has been used frequently to establish drug efflux driven by P-gp. The expression of P-gp on the apical surface of this cell line makes this cell line particularly useful^[37]^. Moreover, the CYP3A4 enzyme is missing in Caco-2 cells. To further elucidate the mechanism of the reversal of docetaxel-mediated drug resistance by ritonavir, the bidirectional permeability of docetaxel was tested in Caco-2 monolayer assays at 5 and 10 µM in the absence and in the presence of the P-gp specific reference inhibitor valspodar and ritonavir applying 60- and 120-minute incubation periods confirming that docetaxel is an *in vitro* substrate of the P-gp transporter [Supplementary Table 1]. Specifically, the bidirectional permeability from Apical to Basolateral (A-B), from Basolateral to Apical (B-A), and ER values of docetaxel in the presence of the inhibitors valspodar and ritonavir were determined. The effect of docetaxel on the monolayer integrity was also investigated by applying low permeability control Lucifer LY in the absence and presence of the docetaxel (data not shown).

In the bidirectional Caco-2 monolayer assay, docetaxel showed active transport as the obtained Papp values in the B-A direction were higher than in the A-B direction and the ER values were > 2 both at 5 and 10 μM. The ER values were 69.5 and 43.9 at 5 μM and 31.8 and 32.7 at 10 μM of docetaxel after 60- and 120-minute incubation time, respectively [Table 8]. The P-gp reference inhibitor valspodar and ritonavir inhibited the active transport of docetaxel, resulting in ERs < 2. These show that docetaxel is transported via P-gp in the B-A direction through Caco-2 monolayers and this drug transport can be inhibited by ritonavir.

**Table 8:** Bidirectional permeability and efflux ratio values for docetaxel and inhibitors across Caco-2 monolayers after 60 and 120 min of incubation

**Compound**	**Concentration**	**Papp A-B** **(× 10^-6^ cm/s)**	**Papp B-A** **(× 10^-6^ cm/s)**	**ER**
**Incubation 60 min**				
Docetaxel	10 μM	0.66 ± 0.02	21.03 ± 0.78	31.80 ± 1.54
Docetaxel + valspodar	10 μM + 10 μM	5.20 ± 0.40	5.39 ± 0.22	1.04 ± 0.09
Docetaxel + ritonavir	10 μM + 50 μM	5.33 ± 0.33	6.62 ± 0.24	1.24 ± 0.09
Docetaxel	5 μM	0.29 ± 0.06	20.27 ± 0.25	69.46 ± 13.24
Docetaxel + valspodar	5 μM + 10 μM	1.61 ± 0.53	4.02 ± 0.09	2.49 ± 0.82
Docetaxel + ritonavir	5 μM + 50 μM	2.87 ± 0.48	5.74 ± 0.08	2.00 ± 0.34
**Incubation 120 min**				
Docetaxel	10 μM	0.91 ± 0.15	29.62 ± 0.80	32.66 ± 5.35
Docetaxel + valspodar	10 μM + 10 μM	8.91 ± 1.33	9.51 ± 0.37	1.07 ± 0.16
Docetaxel + ritonavir	10 μM + 50 μM	10.76 ± 0.10	11.59 ± 0.33	1.08 ± 0.03
Docetaxel	5 μM	0.81 ± 0.06	35.40 ± 1.83	43.85 ± 3.80
Docetaxel + valspodar	5 μM + 10 μM	8.06 ± 1.10	8.58 ± 0.63	1.06 ± 0.17
Docetaxel + ritonavir	5 μM + 50 μM	7.41 ± 1.28	11.83 ± 0.33	1.60 ± 0.28

Data are expressed as mean (*n* = 3) ± SEM for Papp or ± EP for ER. A-B: Apical to basolateral direction; B-A: basolateral to apical direction; Papp: apparent permeability coefficient; ER: efflux ratio.

## DISCUSSION

Even though chemotherapy resistance may have a variety of causes, multidrug resistance is often associated with overexpression of P-gp. Therefore, the relationship between P-gp efflux and multidrug resistance has been extensively studied. Unfortunately, in the clinical setting, the success of drugs targeting multidrug resistance has been limited^[38]^. In previous work, it was shown that ritonavir enhances the bioavailability of oral docetaxel by inhibiting both P-gp and CYP3A4. In the current study, we investigated if adding ritonavir to docetaxel increased antitumor effects in docetaxel-resistant prostate cancer cells. We have determined the single agent activity of docetaxel, ritonavir, and docetaxel plus ritonavir in 15 tumor cell lines. Synergy was observed for the combination of docetaxel and ritonavir in ovarian cancer cell lines 899 and NCI-ADR-RES that have relatively high expression of ABCB1. Interestingly, additive effects between ritonavir and paclitaxel have been observed by Kumar *et al.* in two different ovarian cancer cell lines (MDH-2774 and SKOV-3)^[39]^. The authors suggest a relationship between the inhibition of AKT phosphorylation by ritonavir and the induction of apoptosis, particularly in drug-resistant ovarian cancer. However, in our studies, no synergy between docetaxel and ritonavir was seen in the 22Rv1, DU-145, PC-3, and PC-3M cell lines. This contrasts with the observation by Ikezoe *et al.*, who observed a synergistic effect of docetaxel and ritonavir in DU-145 cells^[12]^. Ikezoe used a similar clonogenic assay in soft agar and reported synergistic effects between docetaxel and ritonavir in the DU-145 cell line at low concentrations of 0.1 nM docetaxel and 1 μM ritonavir, which we also tested. Ikezoe suggested that the synergistic effect seen could be attributed to the inhibition of CYP3A4 expression by ritonavir. However, in the DU-145 proliferation assay that was used, single agent ritonavir showed cytotoxic activity at already a relatively low IC_50_ concentration of 3 μM ritonavir. This has not been confirmed by other groups. In a paper by Gills *et al.*, the anti-proliferative effect of ritonavir in the NCI60 cell line panel was tested, and the IC_50_ value in DU-145 cells was > 20 μM^[40]^. This is more in line with the IC_50_ concentration of 16.20 μM for ritonavir that we observed in our DU-145 clonogenic assay.

To model docetaxel mechanisms of acquired resistance that may occur in humans, we established resistant prostate cancer cell lines via prolonged exposure to docetaxel. Specifically, we have generated resistant sublines of DU-145 (DU-145DOC10) and 22Rv1 (22Rv1DOC8). We failed to generate a docetaxel-resistant subline of a PC-3 cell line, while other groups^[22,33,41]^ have been capable of achieving this. Those studies started the induction of resistance at higher concentrations (at 4 and 5 nM) of docetaxel and used considerably longer exposure cycles during the escalation of docetaxel concentrations.

We applied genome-wide RNA sequencing of the resistant sublines and compared the results with the sequencing data of their respective parental cell lines. We found that in both resistant sublines DU-145DOC10 and 22Rv1DOC8, the most highly up-regulated gene compared to the parental cells was ABCB1; this gene was up-regulated 3287-fold in DU-145DOC10 and 2773-fold in 22Rv1DOC8. These gene expression data were confirmed on the protein level by showing that in the parental cell lines DU-145 and 22Rv1, no detectable amount of the P-gp protein was expressed, whereas significant levels of P-gp expression were observed in the DU-145DOC10 and 22Rv1DOC8 cell lines.

In our experiments, the addition of 10 µM ritonavir reversed docetaxel resistance of the DU-145DOC10 resistant subline, whereas the resistant 22Rv1DOC8 subline was less sensitive to 10 µM ritonavir and 32 µM ritonavir was required to revert docetaxel cytotoxicity as observed in the originator 22Rv1 cell line. A confirmation experiment was done by inhibiting P-gp with elacridar and 0.3 µM was already sufficient to reverse docetaxel sensitivity in DU-145-DOC10 and 22Rv1DOC8 cell lines similar to that in DU-145 and 22Rv1 cell lines. These results are comparable to reports from other groups^[22]^ that used elacridar to revert docetaxel sensitivity in DU-145 and 22Rv1 cells. In DU-145-DOC10 and 22Rv1DOC8 cell lines, strong synergistic effects were seen with the docetaxel/ritonavir combination in contrast to the lack of synergy seen in DU-145 and 22Rv1 cell lines. The same synergistic effects were seen with the combination of docetaxel and elacridar in the DU-145-DOC10 and 22Rv1DOC8 cell lines. Our data strongly suggest that ritonavir reverses docetaxel resistance in docetaxel-resistant prostate cancer cell lines.

Cabazitaxel is claimed to have a lower affinity for P-gp based on preclinical studies, where cabazitaxel was more potent than docetaxel in P-gp-associated docetaxel-resistant cell lines^[42]^. Based on a study by Duran *et al.*, it was believed that cabazitaxel is a weaker substrate for P-gp^[43]^. The level of P-gp (over)expression in several ABCB1 positive cell models used by Duran, however, may differ from the levels in our resistance models (DU-145-DOC10 and 22Rv1DOC8). Moreover, internal data included in the cabazitaxel New Drug Application^[44]^ confirm that cabazitaxel, tested in a Caco-2 bidirectional transporter assay, is indeed classified as a P-gp substrate. Furthermore, Machioka *et al.* using two cabazitaxel-resistant CRPC cell lines, identified increased P-gp expression as a driver for cabazitaxel resistance in PC-3 and DU-145 cells^[45]^. In their study, cabazitaxel sensitivity was restored via knocking down ABCB1. In another study^[46]^, C4-2B and DU-145 derived docetaxel-resistant cell lines conferred cross-resistance to cabazitaxel based on increased P-gp expression. In the same study, elacridar re-sensitized docetaxel-resistant cells to treatment with cabazitaxel. Therefore, it is possible that the efflux of cabazitaxel from docetaxel-resistant prostate cancer cells in our study is inhibited by ritonavir.

In a Caco-2 P-gp transporter assay, the bidirectional (A-B and B-A) permeability and ER values of docetaxel in the presence of the P-gp selective inhibitor, valspodar, as well as of ritonavir were determined. In this model, functional inhibition of P-gp-mediated active transport of docetaxel with ritonavir was clearly shown. These results correspond with data from Wils *et al.* showing that the P-gp-based transport of docetaxel in a Caco-2 cell model was also affected by verapamil^[47]^. This further supports the hypothesis that inhibition of P-gp-mediated drug efflux by ritonavir reverses docetaxel resistance.

After we induced resistance to docetaxel in DU-145 and 22Rv1 prostate cancer cell lines, we demonstrated that combining docetaxel with ritonavir is synergistic in docetaxel-resistant cell lines and reverses resistance to docetaxel most likely by P-gp-mediated inhibition of docetaxel efflux. We saw the same synergistic effects for the combination of cabazitaxel and ritonavir in docetaxel-resistant cell lines. To the best of our knowledge, this is the first study to show that ritonavir is capable of reversing resistance to docetaxel in docetaxel-resistant prostate cancer cells. Acquired resistance is limiting the clinical utility^[48]^ of docetaxel and reversing resistance may have a positive effect on survival outcomes in patients with mCRPC. This provides a strong rationale to apply the same drug combination in the clinic through the further clinical evaluation of an oral docetaxel formulation, co-administered with ritonavir, in patients with mCRPC.

## DECLARATIONS

### Availability of data and materials

The data used to support the findings of this study are available from the corresponding author upon request. The substrate assessment of docetaxel for P-gp in the Caco-2 assay is provided as supplemental data in this journal as Supplementary Table 1.

### Copyright

© The Author(s) 2024.
