# Supplementary material for: Ritonavir reverses resistance to docetaxel and cabazitaxel in prostate cancer cells with acquired resistance to docetaxel
Source: Cancer Drug Resist. 2024 Jan 31;7:3. doi: 10.20517/cdr.2023.136 (PMC10838382; doi:10.20517/cdr.2023.136)
Supplement: Supplementary file 1 [file cdr-7-3-SupplementaryMaterials.pdf]

van der Putten *et al. Cancer Drug Resist* 2024;7:3
DOI: 10.20517/cdr.2023.136

Cancer Drug Resistance

Supplementary Material

Ritonavir reverses resistance to docetaxel and cabazitaxel in prostate cancer cells with acquired resistance to docetaxel

Eric van der Putten[1], Katja Wosikowski[1], Jos H. Beijnen[2], Gábor Imre[3], Colin R. Freund[1]

[1]Modra Pharmaceuticals B.V., Amsterdam 1083 HN, the Netherlands.
[2]Department of Pharmacy & Pharmacology, Netherlands Cancer Institute - Antoni van Leeuwenhoek, Amsterdam 1066 CX, the Netherlands.
[3]SOLVO Biotechnology, Budapest H-1117, Hungary.

Correspondence to: Dr. Eric van der Putten, Modra Pharmaceuticals B.V., Barbara Strozzilaan 201, 1083 HN, Amsterdam, the Netherlands. E-mail: eric.vanderputten@modrapharmaceuticals.com

Supplementary Table 1. Substrate assessment of docetaxel for P-gp in the Caco-2 assay

Compound	Concentration	ER (Average ± EP)	Substrate
**Incubation 60 min**			
Docetaxel	10 µM	31.80 ± 1.54	
+ Valspodar	+ 10 µM	1.04 ± 0.09	Yes
+ Ritonavir	+ 50 µM	1.24 ± 0.09	
Docetaxel	5 µM	69.46 ± 13.24	
+ Valspodar	+ 10 µM	2.49 ± 0.82	Yes
+ Ritonavir	+ 50 µM	2.00 ± 0.34	
**Incubation 120 min**			
Docetaxel	10 µM	32.66 ± 5.35	
+ Valspodar	+ 10 µM	1.07 ± 0.16	Yes
+ Ritonavir	+ 50 µM	1.08 ± 0.03	
Docetaxel	5 µM	43.85 ± 3.80	
+ Valspodar	+ 10 µM	1.06 ± 0.17	Yes
+ Ritonavir	+ 50 µM	1.60 ± 0.28	

ER: Efflux ratio; EP: error propagation.
